# Rift Valley and West Nile Virus Antibodies in Camels, North Africa

**DOI:** 10.3201/eid1712.110587

**Published:** 2011-12

**Authors:** Mehdi El-Harrak, Raquel Martín-Folgar, Francisco Llorente, Paloma Fernández-Pacheco, Alejandro Brun, Jordi Figuerola, Miguel Ángel Jiménez-Clavero

**Affiliations:** Biopharma, Rabat, Morocco (M. El-Harrak);; Centro de Investigación en Sanidad Animal, Valdeolmos, Spain (R. Martín-Folgar, F. Llorente, P. Fernández-Pacheco, A. Brun, M.Á. Jiménez-Clavero);; Estación Biológica de Doñana, Seville, Spain (J. Figuerola)

**Keywords:** Rift Valley fever virus, West Nile virus, African horse sickness, camel, antibodies, carriers, arboviruses, vector-borne infections

**To the Editor:** Different arboviral diseases have expanded their geographic range in recent times. Of them, Rift Valley fever, West Nile fever, and African horse sickness are of particular concern. They are endemic in sub-Saharan Africa but occasionally spread beyond this area. Trade and transport of animals and animal products, along with wildlife movements, are considered the driving factors in the spread of these pathogens.

In wide regions of Africa, 1-humped camels (*Camelus dromedarius*) are valuable livestock appreciated as a meat source and as a means for transportation of goods. Camels are susceptible to infection by Rift Valley fever virus (RVFV), West Nile virus (WNV), and African horse sickness virus (AHSV), although their epidemiologic role in these diseases is uncertain (*1**–**3*). Movements of camels across the Sahara Desert could carry these pathogens to northern Africa. To test this hypothesis, we conducted a serologic survey in 1-humped camels intercepted at different points by the Moroccan Veterinary Services in 2009. The camels were coming from the southeastern part of the Sahara Desert going to the northwest.

Serum samples were obtained in Smara-Laayoune, Dakhla, and Tata (Table). Most samples (71 of 100 total samples) were from male camels. Samples were also grouped by age of the camels (Table). RVFV antibodies were detected by using a competitive ELISA (*4*), and samples yielding positive ELISA results were confirmed by virus-neutralization test. WNV-specific antibodies were detected by ELISA (*5*), and positive results were confirmed by virus-neutralization test. AHSV-specific antibodies were detected by using the ELISA prescribed by the World Organisation for Animal Health.

**Table:** Results of testing of camels for virus antibodies, by location, age group, and sex of camels examined, North Africa, 2009*

Camel characteristic	No. samples	No. positive for antibody			
		RVFV	WNV	AHSV	RVFV and WNV
Origin					
Tata	2	0	0	0	0
Smara-Laayoune	58	13	20	0	11
Dakhla	40	2	9	0	1
Age group, y					
<1	18	1	1	0	1
1–2	25	0	1	0	0
3–5	7	1	3	0	1
6–10	27	6	12	0	10
>10	23	7	12	0	0
Sex					
M	71	10	15	0	7
F	29	5	14	0	5
Total	100	15	29	0	12

*RVFV, Rift Valley fever virus; WNV, West Nile virus; AHSV, African horse sickness virus.

Fifteen of 100 samples were positive for RVFV-specific antibodies by competitive ELISA, all of which were confirmed by virus-neutralization test, with neutralization titers ranging from 40 to 1,280 (geometric mean titer = 229). With regard to WNV antibodies, the ELISA detected 44 positive samples and 1 doubtful sample, of which 29 were confirmed as positive by virus-neutralization test (virus-neutralization test titers ranging from 10 to 640; geometric mean titer = 20). As for AHSV antibodies, none of the samples was positive by ELISA. Prevalence data were analyzed by generalized linear model with locality (Dakhla or Smara), sex, and age as fixed factors. No differences by origin or sex were found in prevalence for WNV (p>0.14) but antibodies were more prevalent in camels >3 years of age (χ^2^ = 14.04, 3 df, p = 0.003). No differences in prevalence of RVFV antibodies were found by sex (p = 0.29), but prevalence was higher in Smara (χ^2^ = 3.74, p = 0.05) and among camels >6 years of age (χ^2^ = 8.37, df = 3, p = 0.04) (Table). We also examined the co-occurrence of antibodies to RVFV and WNV. Of 15 RVFV-positive samples, 12 were also positive for WNV antibodies, and 12 of 29 WNV-positive samples were also positive for RVFV (χ^2^ = 8.37, df = 1, p < 0.05).

Antibodies to 2 zoonotic arboviruses, i.e., RVFV and WNV, were present in camels moving to the northwestern part of the Sahara Desert, and antibodies to AHSV were absent in the populations examined. Despite the higher percentage of seropositivity for WNV than for RVFV, the epidemiologic consequence of RVFV-specific antibodies in this population could be higher than that for WNV antibodies. Camels can act as reservoir hosts for RVFV (*6*) but are unlikely to do so for WNV, which cycles between mosquitoes and wild birds with mammals usually being dead-end hosts. High prevalence of antibodies to RVFV in camels has been described in different sub-Saharan and Sahelian countries (*7**–**9*). Camels have been involved in the spread of disease in some instances (*10*). Immunity to RVFV indicates previous infection. Our results showed that seroprevalence of RVFV was higher among older than younger camels, indicating that contact could have occurred some years ago. Nevertheless, these populations should be monitored for RVFV and other arboviroses because these are known to reemerge under certain circumstances in locations where they have occurred in the past.

The results of this study support that camels moving across the Sahara have contact with RVFV and WNV, and frequently the same animals have been infected by both agents. In a particularly dry environment such as the desert, particular attention should be paid to singular wet areas such as oases. The presence of water in these areas results in an abundance of competent mosquitoes and hosts, which in turn makes these viruses likely to cycle and infect domestic animals such as camels coming to drink and rest.
